# Nitric Oxide and S-Nitrosylation in Cardiac Regulation: G Protein-Coupled Receptor Kinase-2 and β-Arrestins as Targets

**DOI:** 10.3390/ijms22020521

**Published:** 2021-01-07

**Authors:** Gizem Kayki-Mutlu, Walter J. Koch

**Affiliations:** 1 Center for Translational Medicine, Temple University School of Medicine, Philadelphia, PA 19140, USA; gkayki@ankara.edu.tr; 2 Faculty of Pharmacy, Department of Pharmacology, Ankara University, Tandogan, 06560 Ankara, Turkey

**Keywords:** GRK2, β-arrestins, nitric oxide, S-nitrosylation

## Abstract

Cardiac diseases including heart failure (HF), are the leading cause of morbidity and mortality globally. Among the prominent characteristics of HF is the loss of β-adrenoceptor (AR)-mediated inotropic reserve. This is primarily due to the derangements in myocardial regulatory signaling proteins, G protein-coupled receptor (GPCR) kinases (GRKs) and β-arrestins (β-Arr) that modulate β-AR signal termination via receptor desensitization and downregulation. GRK2 and β-Arr2 activities are elevated in the heart after injury/stress and participate in HF through receptor inactivation. These GPCR regulators are modulated profoundly by nitric oxide (NO) produced by NO synthase (NOS) enzymes through S-nitrosylation due to receptor-coupled NO generation. S-nitrosylation, which is NO-mediated modification of protein cysteine residues to generate an S-nitrosothiol (SNO), mediates many effects of NO independently from its canonical guanylyl cyclase/cGMP/protein kinase G signaling. Herein, we review the knowledge on the NO system in the heart and S-nitrosylation-dependent modifications of myocardial GPCR signaling components GRKs and β-Arrs.

## 1. Introduction

G protein-coupled receptors (GPCRs) play important roles in the regulation of cardiac function. Upon agonist stimulation, GPCRs are phosphorylated by GPCR kinases (GRKs) and thereafter associate with β-arrestins (β-Arr), which regulate receptor desensitization and internalization. Nevertheless, activities of these regulatory molecules are augmented after stress/injury and result in excessive signal uncoupling and receptor desensitization which contribute to pathogenesis [1,2]. Thus, inhibition of the pathological upregulation of these myocardial proteins is known to be therapeutic [3,4,5].

An emerging important regulator of GPCR signaling is nitric oxide (NO), which in itself has key cardiovascular regulatory properties [6]. NO modulates cardiovascular function through two different pathways: through activation of soluble guanylyl cyclase (sGC)/cGMP-dependent protein kinase G (PKG) pathway and through a post-translational modification, so called S-nitrosylation, that is the coupling of NO moiety to cysteine residues of target proteins [6,7]. S-nitrosylation mediates many significant cardiac effects of NO [8,9,10]. Important GPCR molecules in the heart including β-adrenoceptors (βARs), GRKs and β-Arr can be modulated by S-nitrosylation that is triggered by NOS-mediated generation of NO [11,12,13,14]. Here, we will review the importance of the NO system in the heart and findings on S-nitrosylation-dependent modifications of the GPCR regulators, GRKs and β-Arrs.

### 1.1. NO: An Essential Signaling Molecule

Nitric oxide (NO) is a gaso-transmitter, which was initially characterized as endothelium-derived relaxing factor (EDRF) [15]. NO is a small molecule and soluble in both aqueous and hydrophobic environments, thus it can easily diffuse across biological membranes which makes it a suitable biological messenger. Despite its simple structure, it is involved in many complex reactions and mediates numerous physiological functions [15,16]. It can exert control on vascular tone, inhibit platelet activity, regulate gene transcription, influence the immune system, affect neuronal development and behavioral responses, regulate cardiac function and finally has activity to fight tumor progression and bacterial infections [16,17].

NO is involved in important biological and physiological reactions which are divided into two categories: direct and indirect [18]. The direct effects are the chemical reactions that allow NO to react with a biological target molecule directly. Indirect effects, on the other hand, require the reaction of NO with oxygen and superoxide to produce reactive nitrogen species (RNS), which will ultimately react with the biological targets [7,18]. Normally, direct effects occur at low concentrations of NO while indirect effects require higher NO concentrations. Indirect effects lead to either nitrosative or oxidative stress [18]. Thus, NO is a vital molecule at physiological concentrations, however its prolonged production can lead to various disease states including inflammation and cancer [19,20].

NO is mainly produced by NO synthases (NOS) or by the conversion of endogenous substances such as inorganic nitrite [10,21]. Three forms of NOS catalyzes the synthesis of NO, NOS1 (neuronal or nNOS), NOS2 (inducible or iNOS) and NOS3 (endothelial or eNOS) [9]. They convert L-arginine and oxygen to L-citrulline and NO in the presence of nicotinamide-adenine-dinucleotide phosphate (NADPH) as co-substrate and tetrahydrobiopterin (BH_4_), flavin adenine dinucleotide (FAD), flavin mononucleotide (FMN) as cofactors [9,22]. NOS generates NO through two steps. The first step is the L-arginine hydroxylation to N^ω^-hydroxyl-L-arginine and the second step is the oxidation of N^ω^-hydroxyl-L-arginine into L-citrulline and NO [23]. NOS is regulated by other molecules such as NADPH and calmodulin (CaM) [24,25].

NOS isoforms differ significantly in their regulation, distribution and activity. nNOS and eNOS are constitutively present in many cell types while iNOS is induced under stress conditions [7]. Enzymatic activity of all isoforms depends on substrate and cofactor availability. Reduced substrate availability was reported to result in the generation of superoxide rather than NO [18]. On the other hand, another substrate oxygen dependency differs among each isoform. While eNOS is less affected by oxygen fluctuations, nNOS is the most sensitive isoform [26]. In case of low O_2_, NOS forms superoxide [27]. Thus, translocation of NOS to another compartment with a different oxygen levels alter its activity [18].

#### 1.1.1. Endothelial Nitric Oxide Synthase

Under basal conditions, eNOS is localized to invaginations of sarcolemma called caveolae [28]. Here, it is maintained in an inactive state as it interacts with caveolin-1 or 3 which are tonic eNOS inhibitors. Upon agonist stimulation, NOS translocates fromthe membrane and start to interact with regulator proteins, CaM and hsp90, as Ca^2+^ levels increase, which in turn leads to enzyme activation [24]. Activated NOS translocates to the cytosol [6]. eNOS can also be activated independently from intracellular Ca^2+^ and induce a long-lasting NO release. Fluid shear stress, stretch, estrogens, vascular endothelial growth factor, insulin and bradykinin are such stimuli that activate the enzyme in this manner [6,29]. eNOS is predominantly expressed in endothelial cells with detectable expression in cardiomyocytes, platelets and some neurons [6,24].

eNOS-derived NO is mainly responsible for vasodilation regulating blood vessel tone. Additionally, it mediates anti-proliferative, anti-aggregation and anti-apoptotic effects of the endothelium. Thus, it is important in preventing atherosclerosis [24]. Decreased eNOS expression may result in endothelial dysfunction. Conversely, increased eNOS is also reported to be associated with a cardiovascular risk due to excess ROS production [30].

eNOS activity is regulated via various modifications including phosphorylation, protein–protein interactions, acetylation, and S-nitrosylation [31,32]. Ser^1177^ is its main activation site while Thr495 is the inhibitory site to induce activity [33,34]. Phosphorylation of Ser^615^, Ser^633^ and Tyr^81^ are also additional sites that stimulates NO synthesis. However, phosphorylation of Ser^114^ and Tyr^657^ decreases NO production through eNOS [6]. Deacetylation of eNOS, on the other hand, decreases NO production due to diminished CaM association [32]. Moreover, in a product feedback mechanism, eNOS is modified by S-nitrosylation on Cys^94^ and Cys^99^ by NO [32].

#### 1.1.2. Inducible Nitric Oxide Synthase

iNOS is barely detected under normal conditions and its expression is stimulated in response to pro-inflammatory or oxidative conditions. iNOS is primarily found in macrophages although its expression levels can be induced in different cell types including cardiomyocytes, neurons, smooth muscle cells and hepatocytes [35]. When it is expressed, it is constantly active and is not regulated by intracellular Ca^2+^ concentrations, in contrast to eNOS and nNOS [6,24]. Its catalytic activity is 100–1000 times higher than the other isoforms, thus maintains a high NO output. NO can inhibit enzymes containing iron such as those involved in mitochondrial electron transport and also interact with DNA of target cells and cause fragmentation. Through these effects, it can act as cytostatic and cytotoxic on invading pathogens or tumor cells, thus iNOS signaling is critical for inflammatory response and the immune system. However, when dysregulated it may also harm healthy cells. iNOS has been implicated in various pathologies including cardiovascular diseases, diabetes, cancer, sepsis and neurodegeneration [36].

#### 1.1.3. Neuronal Nitric Oxide Synthase

The initial characterized NOS isoform, nNOS, is constitutively expressed primarily in nervous system and also cardiomyocytes and vascular smooth muscle cells. It is regulated by Ca^2+^ and CaM and typically is located within the endo- or sarco-plasmic reticulum. Five splice variants of nNOS have been demonstrated: nNOSα, nNOSβ, nNOSµ, nNOSγ and nNOS2 [37]. Diverse subcellular localization of nNOS contributes to various functions. In CNS, nNOS modulates synaptic transmission and thereby plays a role in regulating learning and memory. It has also been implicated in central regulation of blood pressure and peripheral vascular tone modulation [24]. NO released by nNOS containing nitrergic nerves, which innervate smooth muscles in blood vessels, mediates a decrease in vascular tone. In case that eNOS does not function properly, nNOS-mediated vasodilation may become more prominent [38]. nNOS also produces H_2_O_2_ which contributes to vascular relaxation under physiological conditions and its decline evokes endothelial dysfunction [39].

nNOS signaling is also important for cardiac function. It modulates ion regulation involving Ca^2+^ homeostasis and mitochondrial function [37]. To fulfill cardiac functions, its compartmentation is critical in the heart. It is bound to ryanodine receptors (RyR) on sarcoplasmic reticulum (SR) membranes, whereas, under stress conditions it translocates from the SR to the plasma membrane as a protection mechanism against Ca^2+^ overload [37]. nNOS can be regulated by kinases and also CaM [39,40].

#### 1.1.4. Nitric Oxide Signaling

NO mediates two distinct pathways, one mediated by soluble guanylate cyclase (sGC) and another through the direct S-nitrosylation of proteins [6,41]. Classically, upon NO binding to sGC, guanosine triphosphate (GTP) is converted into cyclic guanosine monophosphate (cGMP), which in turn activates cGMP-dependent kinase (PKG) and is hydrolyzed by cAMP phosphodiesterase (PDE). Through this pathway, NO mediates vasodilation, inhibits vascular smooth muscle (VSM) proliferation, diminishes platelet aggregation, vascular inflammation [42] and positive lusitropic effects [43]. Moreover, function and phosphorylation of various cardiac proteins such as RyR2, the L-type calcium channel (LTCC) and phospholamban are affected via the cGMP-dependent pathway [44].

Importantly, and a primary subject of this review, increasing evidence has emerged over the last decade showing that many effects of NO are mediated via a cGMP-independent pathway and one that can affect the activity, cellular localization and regulation through binding partners of several proteins. This is through NO acting on cysteine residues through S-nitrosylases generating S-nitrosothiol (SNO) on target proteins generated a post-translational modification known as S-nitrosylation [9].

### 1.2. NO-Mediated Signaling: S-nitrosylation

S-nitrosylation is defined by the binding of NO on a thiol (-SH) group of cysteine residues forming an SNO molecule. The primary sources of NO for this modification are the three NOS isoforms. Inorganic nitrate and nitrite from endogenous or dietary sources also contribute to NO formation [45]. Transfer of NO to cysteine residues in target protein is mediated by two classes of enzymes: SNO synthases and transnitrosylases [46,47]. While SNO synthases transfer NO from transition metals to cysteine thiol, transnitrosylases transfer NO between SNO proteins [48]. Trans-S-nitrosylation plays an important role in the transmission of NO/SNO signal within different compartments of the cell. For example, although NOS is absent in the nucleus, SNO signaling occurs via trans-S-nitrosylation from different proteins (e.g., GAPDH) in that compartment [47]. Conversely, SNO is removed by denitrosylases including S-nitrosoglutathione reductase (GSNOR) and thioredoxin using NADH and NADPH as electron donors. The equilibrium between S-nitrosylation and denitrosylation pathways is regulated by SNO-protein abundance rather than NO production rate [8].

As cysteine thiol-containing proteins are widely available, such proteins are most likely to be subjected to the regulation via S-nitrosylation. The target protein to be nitrosylated can be simple or complex members such as enzymes, G proteins, transcription factors, transporters and ion channels [49]. As a result of S-nitrosylation, activity, protein interactions, trafficking, localization and degradation of the target protein are affected [50]. For instance, S-nitrosylation may influence kinase substrate specificity (e.g., ASK1, JNK), may modify protein isoforms interacting with one another (e.g., β-arrestins), may convert a protein kinase into a protein nitrosylase (e.g., GAPDH, CDK5, GSK3β) or prevent irreversible oxidation [6,47]. While some proteins are activated via S-nitrosylation (such as dynamin [13], RyR2 [51], β-arrestin2 [13], some others are inhibited such as eNOS [52] and GRK2 [14].

NOS signaling itself, is also affected by S-nitrosylation. S-nitrosylation of sGC was also reported to have diminished NO responsiveness [53]. An essential cofactor for eNOS, BH4 is regenerated by dihydrofolate reductase (DHFR) whose S-nitrosylation is crucial for its stability and thus the maintenance of eNOS coupling [54]. Importantly, decreased levels of SNO proteins have been observed in various disease states involving hypoxia. However, uncontrolled SNO production, termed as nitrosative stress may also contribute to multiorgan failure [8]. SNO content has shown to be increased in septic shock and acute respiratory distress syndrome [55,56].

Although all three NOS isoforms produce NO for S-nitrosylation, each isoform can mediate selective S-nitrosylation of target proteins [57]. Intracellular compartmentalization of NOS is an important determinant in S-nitrosylation specificity [9,58]. eNOS localized within the caveolae is in close proximity to LTCC and modulates ion flux through LTCC S-nitrosylation [9]. eNOS localization on the Golgi apparatus enhances S-nitrosylation of the Golgi proteins [58]. Within the corporal endothelial cells, eNOS is reported to be colocalized with GSNOR that catabolizes S-nitrosylated proteins. Thus, eNOS plays a role in the S-nitrosylation/denitrosylation feedback loop in this tissue [59]. Similarly, nNOS/GSNOR interaction due to their colocalization maintain skeletal muscle homeostasis [60]. Further, nNOS located in sarcoplasmic reticulum (SR) targets the proteins in this compartment such as RyR1 (in skeletal muscle) and RyR2 (in cardiac muscle) whose S-nitrosylation alters channel opening probability [35]. Moreover, iNOS-mediated inhibitory S-nitrosylation of mitochondrial proteins was demonstrated to initiate apoptotic programs [61]. iNOS-regulated S-nitrosylation has also been shown to lead to diminished ER function in obesity [62] and mTOR pathway-related proliferation in melanoma [63]. Adaptor proteins are claimed to contribute specific substrate cognition by different NOS isoforms [57]. PSD-95 [64] and CAPON [65] were reported to direct nNOS to the target protein to be nitrosylated. S100A8/A9 was shown to act as a scaffold for iNOS binding to GAPDH [57].

### 1.3. Role of S-Nitrosylation in the HEART

In the cardiovascular system, there are a wide range of factors that undergo S-nitrosylation [9]. Proteins that are functional in Ca^2+^ homeostasis, mitochondria, hemoglobin, as well as sarcomeric proteins and ion channels regulating contractility are targets for S-nitrosylation by both endogenously produced NO as well by exogenous donors [6,50]. Within the heart, there is a balance between S-nitrosylation and denitrosylation regulated in concert with NOS and GSNOR. GSNOR, which controls SNO levels by promoting denitrosylation, is important for cardiac function through regulating vascular tone and β-AR-activated contractility [66]. GSNOR deficiency was demonstrated to increase regeneration post myocardial infarction supporting a cardioprotective role for S-nitrosylation [67]. Conversely, GSNOR overexpression is also shown to protect the heart against sepsis-induced myocardial depression [68] and chronic β-AR stimulation [69].

S-nitrosylation regulates Ca^2+^ handling proteins working in concert with phosphorylation and plays an essential role cardiac function. S-nitrosylation of RyR2 augments open channel probability, thus Ca^2+^ release and catecholamine-induced contractility [51,70]. SERCA S-nitrosylation increases Ca^2+^ uptake through increased activity [71], whereas LTCC S-nitrosylation inhibits its function of ion transport [10,72]. Within cardiomyocytes, nNOS located in SR mediates RyR2 and SERCA S-nitrosylation [51,70,73]. In failing hearts, nNOS is reported to be transported to the plasma membrane [25] and this translocation was shown to regulate Ca^2+^ handling [74]. On the other hand, the caveolae-resident isoform, eNOS, S-nitrosylates LTCC [72]. Moreover, S-nitrosylation of phosholamban and troponin C functioning in parallel with phosphorylation, also plays essential role in cardiac function [69].

More ion channels participating in excitation–contraction coupling are subject to S-nitrosylation. For instance, S-nitrosylation of slowly activating delayed rectifier potassium channel (I_Ks_) channel [75], ultrarapid component of the delayed rectifier (I_Kur_) [76], transient outward K^+^ current [77] were all shown to be regulated by S-nitrosylation. Moreover, nNOS-mediated S-nitrosylation of voltage-gated sodium channel was reported to cause late sodium current [78]. Sarcomeric proteins are also subject to S-nitrosylation which results in myofilament desensitization to Ca^2+^ and depression of contractile activity [79].

Due to the high amount of nitrosylating agents and cysteine consisting proteins in mitochondria, proteins in that compartment are also subject to S-nitrosylation which mostly inhibits their activities. S-nitrosylated complex I was shown to mediate attenuated ROS production during I/R [33]. S-nitrosylated cytochrome c oxidase [80] and F1F0ATPase [81] has inhibited activity resulting in diminished oxygen and ATP consumption, respectively. SNO of mitochondrial permeability transition pore (MPTP) blocks its opening and thus prevents mitochondrial dysfunction leading to cardiomyocyte death [82]. On the other hand, SNO augments parkin [83] and α-ketoglutarate dehydrogenase [71] activities resulting in the modulation of mitochondrial degradation and prevention of oxidative stress. S-nitrosylation of electron chain proteins were demonstrated to be increased in heart failure and mediate decreased ATP production [84].

Critical oxygen-responsive elements in cardiovascular system are also regulated via S-nitrosylation. Alveolar ventilation and perfusion, cardiac muscle performance and microcirculatory blood flow are modulated through this posttranslational modification that maintains a crosstalk between NO and oxygen delivery [8]. Reduced O_2_ delivery in hypoxia and anemia activates hypoxia inducible factor 1 (HIF-1) that regulates hypoxic adaptation transcriptionally through NO-mediated S-nitrosylation [85]. A cysteine residue within hemoglobin (Hb) also exhibits S-nitrosylation to sense oxygen and thereby regulate vascular tone [86]. Moreover, S-nitrosylation of β_2_-ARs modulate responses in multiple organs, including airway relaxation in lungs, augmentation of performance in heart and skeletal muscle [8].

Like other post-translational modifications, S-nitrosylation may also modify protein function through altering its location and binding partners [48]. For instance, SNO of GAPDH induces its translocation to the nucleus through binding to Siah1 (and E3 ubiquitin ligase) and this translocation initiates [10]. Moreover, GOSPEL (GAPDH’s competitor of Siah1 Protein Enhances Life) is also subject to S-nitrosylation resulting in diminished GAPDH binding to Siah1 and thus decreased apoptosis [48]. S-nitrosylation competes with other posttranslational modifications including oxidation and thus prevents thiol(s) from further oxidation acting as a shield against excessive oxidative stress [87].

Taken together, SNO may exhibit protective or detrimental effects according to level, location and target protein [10,64,88]. Hyper-nitrosylation mediated by the β-AR results in SR Ca^2+^ leak and decreased contractility [66]. Similarly, increased denitrosylation prevents persistent β-AR activation-mediated LV remodeling [69]. On the other hand, GSNOR deficiency was also demonstrated to improve SR Ca^2+^ leak [89] and myocardial injury [81]. Moreover, in long QT syndrome, a mutation in syntrophin causes aberrant S-nitrosylation of sodium channels and therefore increased late Na^+^ currents leading to cardiac dysfunction [78]. In addition, due to the fact that SNO is a redox-dependent reaction, increased oxidative stress augments NO consumption and thereby decreases protein SNO. Additionally, NOS uncoupling leads to ROS production [48]. Therefore, changes in NOS signaling resulting in dysregulated S-nitrosylation are proposed as a risk factor for cardiovascular diseases [9,73]. Conversely, SNO can also modify ROS generation and control redox-active enzymes [10].

### 1.4. Role of S-Nitrosylation in GPCR Signaling

GPCRs play important roles in a wide variety of physiological and pathological processes in the cardiovascular system through transducing signals through G proteins. GPCRs are important targets of S-nitrosylation and NO regulation. Critical GPCRs that regulate cardiac function include β-ARs where ligand binding leads to a conformational change in the receptor resulting in the conversion of GDP to GTP. This exchange dissociates G protein into two units: GTP-bound Gα subunit and Gβγ complex. Both interact with numerous effectors and mediate different effects [1,2,5].

GPCR activation can initiate regulatory feedback loops to control GPCR signaling through receptor phosphorylation by GRKs (homologous desensitization) or by second messenger-activated protein kinases (heterologous desensitization) [4,12]. In addition to receptor phosphorylation, NO, which is produced following GPCR activation, can also control GPCR signaling through S-nitrosylation and leads to the inhibition of G protein coupling [19].

β-ARs are the predominant cardiac GPCR that regulate cardiac function [2]. They play critical roles both in physiological and pathological conditions and are common targets for treating cardiovascular disorders. All β-AR subtypes (β1-, β2-, and β3-ARs) are expressed in the heart but couple to different signaling pathways. β1- and β2-ARs primarily activate Gs-adenylyl cyclase-cAMP pathway and mediate myocardial contractility in response to sympathetic nervous system (SNS) activation [1]. β2-ARs are also known to have an additional coupling to Gi through a PKA-dependent G-protein switching mechanism [90]. While excessive β1AR stimulation exerts detrimental effects via promoting myocyte death and activation of adverse signaling pathways, β2ARs have been shown to mediate myocyte survival and have cardioprotective effects [91]. This survival signaling of β2ARs appears to involve increased eNOS activity [92]. The third subtype, β3ARs couples to both Gs and Gi and mediates negative inotropy. Although it is expressed at low levels in the heart, it is a major stimulator of eNOS and nNOS thereby providing cardioprotection [93].

**Figure 1:**
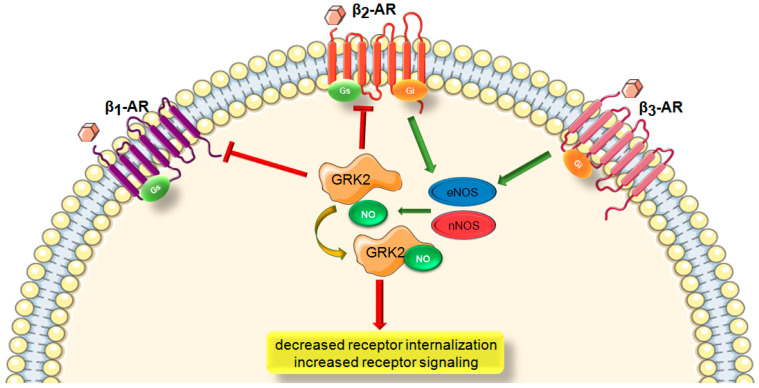
Schematic representation depicting the current understanding of the interaction between β-AR-signaling and GRK2 S-nitrosylation. β-AR, β-adrenergic receptor; G-protein subtypes (Gs or Gi); GRK2, G protein-coupled receptor kinase 2; NO, nitric oxide; eNOS, endothelial nitric oxide synthase; nNOS, neuronal nitric oxide synthase. Green arrow is used to indicate a stimulatory mechanism involved while red bar-headed line indicates an inhibitory mechanism.

GPCRs are regulated by a dynamic and finely tuned activation–deactivation mechanism. As their sustained activation has detrimental effects, regulated termination is important to maintain normal function [2]. G protein-coupled receptor kinases (GRKs) prevent further receptor activation and suppress signaling. GRK phosphorylates serine/threonine residues of the receptor and facilitates β-Arr recruitment to the receptor [1,4]. Nevertheless, in contrast to classical GRK-mediated phosphorylation of β1 and β2-ARs, β3-ARs are not subjected to this regulation. Thus, they are resistant to downregulation and even are upregulated after cardiac injury which makes them uniquely important in the development of cardiac pathologies [7]. Since β3-ARs robustly couple to NOS-NO signaling, they are critical to NO regulation and most probably S-nitrosylation in the heart as well (Figure 1).

GPCRs and β-arrestins regulate GPCR desensitization and internalization through various posttranslational modifications including ubiquitination [94] and phosphorylation [95]. GRK2 phosphorylation of β1- and β2-ARs upon agonist stimulation and subsequent β-arrestin recruitment followed by receptor internalization have also been shown to be regulated by S-nitrosylation [14]. S-nitrosylation-mediated GPCR regulation represents a major mechanism through which NO exerts its various effects [11]. For example, this modification inhibits GRK2 activity and potentiates cAMP signaling and decreases internalization.

NO also can directly regulate GPCR activity [19]. NO donors were demonstrated to inhibit G protein coupling of muscarinic [96] and bradykinin [97] receptors. Treatment with the NO donor, GSNO, was reported to inhibit α1-AR-mediated vasoconstriction due to decreased ligand binding and increased S-nitrosylation of the receptor [98]. Likewise, AT-1 receptor S-nitrosylation decreased ligand binding in response to NO-donor treatment [99]. NO donor treatment also can decrease β2-AR-mediated cAMP accumulation [100] and receptor downregulation while in GSNOR knockout mice, β2-AR expressions were found to be increased [14].

### 1.5. Role of S-Nitrosylation in GRK Signaling

GRKs are key molecules in the desensitization and downregulation of ligand-occupied GPCR activity. Depending on the receptor and the tissue, different GRK subtypes are involved, for instance GRK2 and GRK5 are the most prominent ones in the heart [101,102]. They are the primary regulators of β-AR desensitization in response to catecholamines. Under high catecholamine levels, as in the stressed or failing heart, expression and activity of GRK2 and GRK5 are increased. This prolonged rise underlies in the pathological β-AR downregulation and resistance resulting in cardiac dysfunction [103,104].

In addition to canonical functions of GRKs, they also mediate distinct non-GPCR effects in the heart and emerge as a pleiotropic molecule interacting with many non-GPCR interactomes and phosphoproteomes [1]. GRK2 binds to structural proteins such as β-tubulin and HDAC6 and modulates cytoskeletal functions [105,106]. GRK2 also interacts with heat shock protein 90 resulting in mitochondrial translocation of GRK2 under stress, which increases oxidative stress and dysregulates metabolism [107]. Insulin receptor substrate 1 (IRS1) was shown to be another GRK2 substrate, resulting in reduced glucose uptake and thereby insulin resistance [108]. Moreover, CaM binding to GRK5 induces its translocation to the nucleus where it phosphorylates HDAC5 and binds to NFAT promoting pathological hypertrophy [109].

GRK activity is regulated by post-translational modifications and protein–protein interactions [1,19]. PKA- or PKC-mediated phosphorylation of GRK2 at serine residues S685 or S29 mediates its translocation to the membrane where it phosphorylates GPCRs leading to receptor desensitization [110,111]. GRK2 is also phosphorylated by extracellular signal-regulated kinases [28] at serine residue S670 and by Src at tyrosine residues (Y13, Y86, Y92) resulting in reduced activity [106,112]. Additionally, GRK5 activity is also decreased by PKC phosphorylation at serine residues S572, S566, and S568 [113]. On the other hand, GRK activity is also regulated by GRK-interacting proteins. Caveolin 1 or 3 binding to GRK2 and CaM binding to GRK5 inhibit their activities [114,115].

**Figure 2:**
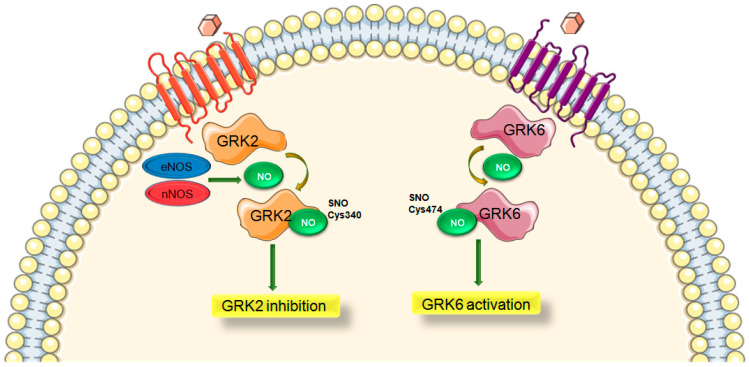
Differential regulation of GRK subtypes via S-nitrosylation. GRK, G protein-coupled receptor kinase; NO, nitric oxide; eNOS, endothelial nitric oxide synthase; nNOS, neuronal nitric oxide synthase. Green arrow is used to indicate a stimulatory mechanism involved.

Moreover, a cysteine residue of GRK2 in position 340 (C340) has been shown to be S-nitrosylated both endogenously and exogenously [14]. While Cys340 is the primary regulatory site, it is also suggested that additional Cys residues may also be subjected to S-nitrosylation resulting different effects of GRK2 activity by this modification [14]. Inhibitory GRK2 S-nitrosylation confirms the prior findings showing that NO/SNO can promote GPCR signaling [116,117] since GRK2 inhibition will prevent desensitization of receptors. GRK6 is also reported to be S-nitrosylated in an age-dependent manner resulting in enhanced kinase activity and this modification was suggested to contribute Parkinson’s disease [118] (Figure 2).

Importantly, GSNOR-deficient mice and mice treated with GSNO have been demonstrated to exhibit improved βAR signaling and expression following continuous ISO exposure [14]. Endogenous or exogenous SNOs were shown to inhibit β-arrestin recruitment and GRK2-mediated receptor phosphorylation and desensitization [14]. Inhibitory GRK2 S-nitrosylation confirms the prior findings that NO/SNO can promote GPCR signaling [116,117]. Additionally, a knock-in (KI) mouse model which has a mutation where native GRK2 Cys340 is replaced with Ser (GRK2-C340S) was shown to exert GRK2 over-activity that leads to increased ischemic injury in the heart [12]. Aged GRK2-C340S KI mice with a global loss of SNO regulation on GRK2 activity have also been demonstrated to present more pronounced hypertrophy over time compared with age-matched controls [119].

Moreover, inhibition of GRK2 via S-nitrosylation was shown to depend on eNOS. Binding of GRK2 to eNOS leads to the inhibition of both. Under stress, increased levels of GRK2 inhibit eNOS resulting in vasoconstriction and remodeling. Conversely, eNOS-mediated GRK2 inhibition enables β-AR signaling therefore improves cardiac function [12]. This relationship between GRK2 and eNOS results in elevated GRK2 activity decreasing eNOS activity or vice versa increased NO bioavailability results in inhibited GRK2 activity via S-nitrosylation acting as an endogenous GRK2 inhibitor. On the other hand, GRK2 was also demonstrated to be constitutively S-nitrosylated in both eNOS and nNOS expressing cells [14] but there is no information about the in vivo effects of different NOS isoforms on GRK2 S-nitrosylation.

Interestingly, S-nitrosylation has opposing effects of GRK2-mediated desensitization and dynamin-mediated internalization [120]. Internalization of GPCRs, which are either in clathrin-coated pits or in caveolae, are regulated by dynamin [121]. This large GTPase binds to eNOS and interacts through S-nitrosylation. This modification augments GTPase activity of dynamin and facilitates fission of vesicles from the membrane [120]. In the absence of a ligand coupled to the receptor, dynamin is found in the cytosol bound to eNOS. Following to the binding of an agonist, dynamin is nitrosylated by eNOS and recruited to the invaginated vesicle on the membrane. Vesicle scission from the membrane and receptor internalization are enhanced as a result [19].

### 1.6. Role of S-Nitrosylation in β-Arrestin Signaling

Among the four-member arrestin family, β-Arr1 and β-Arr2 are well known multi-functional scaffold proteins that have roles in internalization and desensitization of GPCRs. They bind to agonist-occupied, GRK-phosphorylated GPCRs where they inhibit further G protein-coupling leading to decreased responsiveness, known as desensitization and also promote the binding to clathrin-based vesicles [101,122]. Subsequently, their actions can be controlled by dynamin [13]. Some receptors (class A receptors, e.g., β2-AR) only recruit β-Arr2 transiently and translocate into clathrin-coated pits where β-Arr2 disassociates. They internalize afterwards and recycle to the membrane immediately. However, class B receptors (e.g., AT1AR) recruit both βarr1 and β-Arr2 and form stable complexes where they internalize together and are targeted to endosomes for degradation [101,123]. Nevertheless, β1-ARs exhibits a distinct pattern in which β-Arr2 briefly encounters with the activated receptor and then is localized in clathrin-coated pits where they activate ERK pathway. β1-ARs do not colocalize with β-Arr2 in internalized structures [123].

β-Arrs are critical elements in GPCR signaling since they not only mediate desensitization and internalization but also function as transducers activating various pathways independent of G proteins. For instance, they interact with proto-oncogene Src (c-Src) resulting its recruitment to the activated receptor and activation of extracellular signal-regulated kinase (ERK1 or 2) [124]. β-Arrs can scaffold c-Jun amino-terminal kinase (JNK) and ERK1/2 mitogen-activated protein kinase (MAPK) signaling elements [101]. They also inhibit NF-KB (nuclear factor kB)-targeted gene expression [125]. Collectively, β-Arrs may start an additional signaling from their target receptor [19].

β-Arrs also degrade second messengers and limit their signals [19]). For example, they terminate activated Gs-coupled GPCR-mediated-cAMP signals through its degradation following the interaction with phosphodiesterase (PDE4D) [126]. Upon stimulation of Gq-coupled muscarinic receptors, β-Arrs binds to diacylglycerol kinase (DGK) that uses diacylglycerol (DAG) as a substrate and ceases its signaling [127]. Moreover, ligand-occupied D2 dopaminergic receptors recruit β-Arr2 bound to Akt and phosphatase PP2A leading to the inactivation of Akt [128].

Binding of β-Arr to the receptor results in a conformational change which allows it to communicate with signaling intermediates [129]. Depending on the ligand, β-Arr prefers binding to activated GPCR or undergoing translational modifications such as phosphorylation [130], ubiquitination [94] and also S-nitrosylation as emerging evidence shows [13]. Ubiquitination is essential for β-Arr-mediated endocytosis. Ubiquitination of the receptor and of β-Arr, catalyzed by E3 ubiquitin ligases, regulates receptor degradation [94]. On other hand, crosstalk between S-nitrosylation and phosphorylation also modulates β-arrestin function. Both isoforms (β-arrestin1 and β- arrestin2) are either phosphorylated or nitrosylated at multiple loci [47].

A given ligand can interact with either G protein or β-Arrestin leading to different cardiac consequences. The concept known as “biased signaling” describes the ability of different ligands to activate distinct signaling events of one GPCR [2]. Recently, it was reported that biased signaling is controlled by S-nitrosylation of β-Arr [11]. G protein vs. β-Arr biased GPCR is determined by which GRK phosphorylates the receptor or whether β-Arr is S-nitrosylated. In other words, nitrosylation is biased to G protein-independent signaling [11].

S-nitrosylation of β-Arr1 vs. β-Arr2 mediate distinct effects due to peculiar sites specific to the subtype. β-Arr2 was demonstrated to be S-nitrosylated on Cys410 by eNOS and mediates receptor internalization [13] whereas dephosphorylation of Ser412 of β-Arr1 mirrors this effect [130]. For instance, β2AR stimulation activating eNOS promotes β-Arr2-eNOS interaction and thus β-Arr2 S-nitrosylation. This modification leads to disassociation of β-Arr2 from eNOS and its association with clathrin and AP-2 facilitating β2AR trafficking [13]. β-Arr2 also associates with iNOS resulting in augmented NO production. Activated bradykinin receptor 1 triggers β-Arr2-ERK-iNOS signaling and thereby iNOS-mediated NO production [131].

Moreover, β-Arr1/2 were found to be nitrosylated by nNOS and iNOS at Cys251/253. This modification results in the suppression of β-Arr-mediated canonical function and independent β-Arr2 effects [11]. This site is known to promote G protein-biased signaling and prevent SNO augmenting β-Arr signaling and βAR desensitization. Additionally, S-nitrosylation of both β-Arr1/2 were found to be enhanced in heart failure. Thus, S-nitrosylation of β-Arr can be considered a unique feature of heart failure.

**Figure 3:**
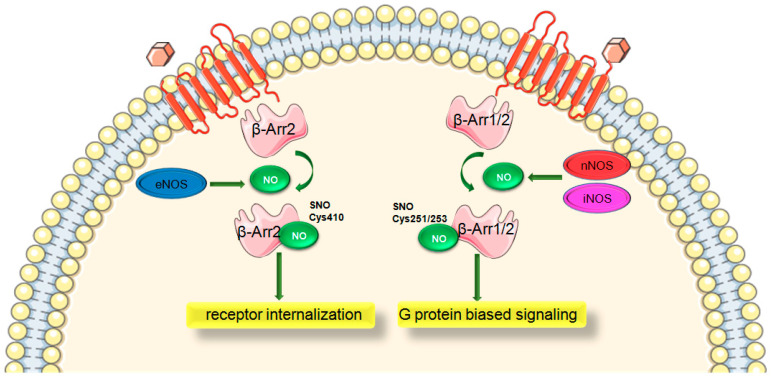
Differential regulation of β-arrestins via S-nitrosylation. β-Arr, β-arrestin; NO, nitric oxide; eNOS, endothelial nitric oxide synthase; nNOS, neuronal nitric oxide synthase; iNOS, inhibitory nitric oxide synthase. Green arrow is used to indicate a stimulatory mechanism involved.

Collectively, all three NOS isoforms may bind to β-Arr1 or 2 but result in distinct signaling (Figure 3). While n/iNOS-regulated S-nitrosylation of β-Arr1/2 inhibits recruitment to the receptors, promotes G protein signaling and modulates desensitization, S-nitrosylation of β-Arr2 by eNOS at Cys410 leads to conformational changes that augment receptor internalization [11,13]. Mice with a mutation of n/iNOS SNO site on β-Arr2 (β-Arr2-Cys253) were shown to develop cardiac dysfunction along with β-AR downregulation upon aging or pressure-overload [11]. Aging is associated with altered NOS expressions and S-nitrosylation states. Old mice were shown to have increased nNOS and iNOS expressions (while eNOS decreased) in heart and lung tissues. S-nitrosylation of β-Arr1 was also enhanced, consistent with S-nitrosylation of Cys251 by nNOS and iNOS. Dimerization of β-Arr2 was also augmented along with a rise in S-nitrosylation of site Cys253 [11]. On the other hand, pressure overload-induced heart failure resulted in significantly impaired cardiac parameters in mutant β-Arr2-C253S mice where S-nitrosylation of β-Arr2 was greatly diminished. S-nitrosylation of Cys 253 was shown to be necessary to maintain β-AR inotropic and chronotropic effects in failing heart. These findings demonstrate that S-nitrosylation of Cys 253 by n/iNOS is cardioprotective through preventing β-AR desensitization and downregulation. Additionally, β-Arr expressions were increased in C253S mice, representing that S-nitrosylation is an important mechanism to suppress β-Arr function. Nitrosylated β-Arr2 appears to regulate HIF-1 and p53 axis promoting adaptive cardiac angiogenesis in heart failure [67].

## 2. Conclusions

Over the last two decades, key myocardial molecules, GRK2 and β-Arrs, affecting GPCR signaling have been shown to be profoundly affected by S-nitrosylation. GRK2 is shown to be subjected to SNO regulation by both eNOS and iNOS, whereas β-Arr2 is S-nitrosylated by distinct NOS isoforms resulting in various consequences. Thus, the desensitization of GPCRs is greatly affected by the changes of all three NOS isoforms. Moreover, it has been demonstrated that β-Arr vs. G protein-biased signaling of GPCRs appears to be determined by which GRK phosphorylates a receptor and whether β-Arr is S-nitrosylated (nitrosylation biases to G protein independent signaling). Together, these findings support an insight: GPCR stimulation is coupled to S-nitrosylation of multiple GPCR components and regulates transduction. Cardiac function thus is not only determined by SNS tone, but also independently by NO bioactivity.

Importantly, these GPCR signaling regulators, GRK2 and β-Arrs, are known to contribute to pathological signaling in cardiac injury and new data demonstrate that S-nitrosylation is also altered in the failing heart. Therefore, it is important to understand cardiac consequences of SNO regulation of key GPCR signaling pathways in the heart. Since the traditional view is that the beneficial effects of eNOS are limited by being downregulated in the heart after stress while iNOS is upregulated mediating negative signaling, there is an imbalance in this system that now needs to be taken into account regarding a balance on all of NO’s targets, especially SNO protein targets. For instance, lack of eNOS-derived NO may cause heart dysfunction due to loss of endogenous inhibition of this pathological molecule in heart failure, while lack of iNOS-derived NO may contribute to heart failure via S-nitrosylation of β-Arr2. The coupling of GPCRs to all three NOSs in the heart as well as SNO in cardiac dysfunction and cardioprotection needs to be evaluated. Investigation of responsible NOS isoforms and the specific regulatory events will be critical to elicit new targets with potential therapeutic uses in heart failure and other cardiac disorders.

## Abbreviations

GPCR	G protein-coupled receptor
GRK	G protein-coupled receptor kinase
β-Arr	β-Arrestin
NO	Nitric oxide
eNOS	Endothelial nitric oxide synthase
nNOS	Neuronal nitric oxide synthase
β-AR	β-Adrenergic receptor
SNO	S-nitrosylation
ROS	Reactive oxygen species
